# Effect of automaticity induced by treadmill walking on prefrontal cortex activation and dual-task performance in older adults

**DOI:** 10.1371/journal.pone.0287252

**Published:** 2023-08-03

**Authors:** Chang Yoon Baek, Hyeong Dong Kim, Dong Yup Yoo, Kyoung Yee Kang, Jang Woo Lee

**Affiliations:** 1 Department of Physical Therapy, College of Health Science, Korea University, Seoul, Republic of Korea; 2 Department of Rehabilitation medicine, National Health Insurance Ilsan Hospital, Ilsan, Republic of Korea; Air University, PAKISTAN

## Abstract

As individuals age, they may experience a decline in gait automaticity, which requires increased attentional resources for the control of gait. This age-related decline in gait automaticity has been shown to contribute to higher prefrontal cortex (PFC) activation and lower dual-task performance during dual-task walking in older adults. This study is to investigate the effect of treadmill walking on PFC activation and dual-task performance in older adults. A total of 20 older adults (mean age, 64.35 ± 2.74 years) and 20 younger adults (mean age, 30.00 ± 3.15 years) performed single- and dual-task walking in overground and treadmill conditions. A wearable functional near-infrared spectroscopy and gait analyzer were used to analyze PFC activation and dual-task performance, respectively. To determine the dual-task (gait and cognitive) performance, the dual-task cost (DTC) was calculated using the following formula: (single-task − dual-task)/single-task × 100. In both groups, dual-task treadmill walking led to reduced PFC activation and reduced DTC compared to dual-task overground walking. Furthermore, despite a higher DTC in gait variability, correct response, total response, response index and a higher error score in older adults than in younger adults during overground walking, there was no difference in treadmill walking. The difference in PFC activation between single- and dual-tasks was also observed only in overground walking. Performing dual-task walking on a treadmill compared to overground walking results in different levels of dual-task performance and PFC activity. Specifically, older adults are able to maintain similar levels of dual-task performance as younger adults while walking on a treadmill, with reduced PFC activation due to the automaticity induced by the treadmill. Therefore, older adults who exhibit low dual-task performance during overground walking may be able to improve their performance while walking on a treadmill with fewer attentional resources.

## Introduction

Aging leads to degradation of physical and cognitive functions [1]. Typically, aging-related changes include decreased gait speed, reduced gait automaticity, and deficits in executive function, [2, 3] which are correlated with alterations in brain regions, such as loss of frontal-lobe gray matter volume and basal ganglia abnormalities [4, 5]. These negative effects are more easily observed in dual-task walking requiring more gait automaticity compared to single-task walking, indicating dual-task interference (DTI) [6]. Hence, dual-task walking could be used to evaluate decreased executive function, reduced gait automaticity, or risk of falling with aging [6, 7]. Brain behavioral studies using functional near-infrared spectroscopy (fNIRS) have reported higher prefrontal cortex (PFC) activation during dual-task walking involving executive functions compared to single-tasking [1, 8]. The negative effects of aging (deficit of gait automaticity) have caused excessive PFC activation, which was compensatory mechanism for automaticity indicating over-recruitment of cognitive resources and reduced efficiency of PFC [3, 9, 10]. That is, higher PFC activation observed in older adults reflects the need for more attentional resources to control gait and compensate for the reduced gait automaticity [1, 9, 11, 12]. Furthermore, since aging-related changes in the PFC occur rapidly, [5] we considered that neural mechanisms in dual-task performance may differ between older and younger adults. However, some brain imaging studies have shown less PFC activation in older adults during dual-tasking, with condition factors (overground and treadmill walking set-ups) and age considered as factors possibly causing these conflicting findings [1, 13, 14]. Although the difference between walking conditions (overground and treadmill) has been raised as a problematic factor, [15–17] findings on both treadmill and overground walking have been generalized [18–22]. To our knowledge, studies addressing walking condition differences and comparing brain activation in older adults to younger adults are lacking. In addition, several studies on dual-task performance have reported different gait mechanisms for overground and treadmill walking [16, 17]. Treadmill walking led to decreased PFC activation and increased gait automaticity in patients with Parkinson’s disease compared with overground walking [23]. When assessing dual task performance in older adults, it is essential to consider environmental factors. This approach can provide a comprehensive evaluation and targeted intervention strategies that account for the unique challenges posed by different environments in older adults with age-related changes [24, 25]. This is the first study to determine and compare PFC activation and dual-task performance during dual-tasking under different conditions (overground vs. treadmill walking) among younger and older adults. Therefore, we aimed to determine 1) whether the treadmill walking condition leads to lower PFC activation and higher dual-task performance than the overground walking condition and 2) whether older adults have higher PFC activation and lower dual-task performance than younger adults. Based on the following evidence showing that gait automaticity induced by treadmill reduced the attention required for the dual-task performance, [16, 23] we hypothesized that 1) treadmill walking would lead to lower PFC activation and higher dual-task performance than overground walking, 2) older adults would have higher PFC activation and lower dual-task performance than younger adults, and 3) there would be a difference between older and younger adults on PFC activation and dual-task performance in overground walking, but not in treadmill walking. Therefore, the results of this study will provide an information in designing an appropriate dual-task evaluation and intervention for older adults.

## Methods

### Participants

This was a cross-sectional study in which 40 right-handed adults were recruited and evaluated from April 29 to June 14, 2021. Among them, 20 were older adults (mean age, 64.35 ± 2.74 years) and 20 were younger adults (mean age, 30.00 ± 3.15 years). This study was approved by the National Health Insurance Service of the Ilsan Hospital Institutional Review Board (NHIMC 2021-03-042-008) and was conducted in a public hospital in accordance with the Declaration of Helsinki. The study was registered in the Clinical Trial Registry of Korea (https://cris.nih.go.kr; no. KCT0006151, 05/20/2021). All participants provided written informed consent before the evaluation. The inclusion criteria were as follows: 1) adults aged between 20 and 40 years or between 60 and 80 years and 2) no ambulatory assistive devices in daily living activities. The exclusion criteria were as follows: 1) Korean version of the Montreal Cognitive Assessment (K-MoCA) score ≤ 17, indicating dementia,(9) 2) orthopedic disorders; 3) neurological disorders; 4) cardiovascular disease; 5) endocrinological disease; 6) psychiatric disease; and 7) poor understanding of the assessment process.

### Screening measure

Initially, the K-MoCA was used to evaluate global cognitive function [26]. Additionally, because the executive function was associated with dual-task gait ability, [27] the executive function was assessed using Trail Making Test B (TMTB), in which the time it took for the participant to connect the numbers and letters alternately (1, A, 2, B,…L, 13) was recorded [28]. Especially for older adults, the Short Physical Performance Battery (SPPB) with scores of 0–12, which included gait, sit-to-standing functions, and standing balance, was used to evaluate the physical performance of older adults [29]. Furthermore, the Geriatric Depression Scale (GDS) with scores of 0–30 and Falls Efficacy Scale-International (FES-I) with scores of 14–64 were used to assess depression and fear of falls, respectively, [30, 31] which can affect the dual-task performance in older adults (i.e., higher scores indicate negative effects) [10]. Retrospective falls within 6 months were also collected with interview. The subjects’ preferred treadmill walking speed was measured first by applying an initial speed of 2.0 km/h and adding increments of 0.1 km/h, then by decreasing the speed from 6.5 km/h, which was determined based on previous findings [17]. After 3 trials, the preferred walking speed on treadmill was determined. The appropriate speed for obtaining the gait automaticity in treadmill walking was self-selected walking speed [32]. Furthermore, the subjects’ preferred overground walking speed was also recorded during the assessment of the gait task described below, and there was no difference in speed between walking conditions (P >0.05).

### Procedure

**Fig 1:**
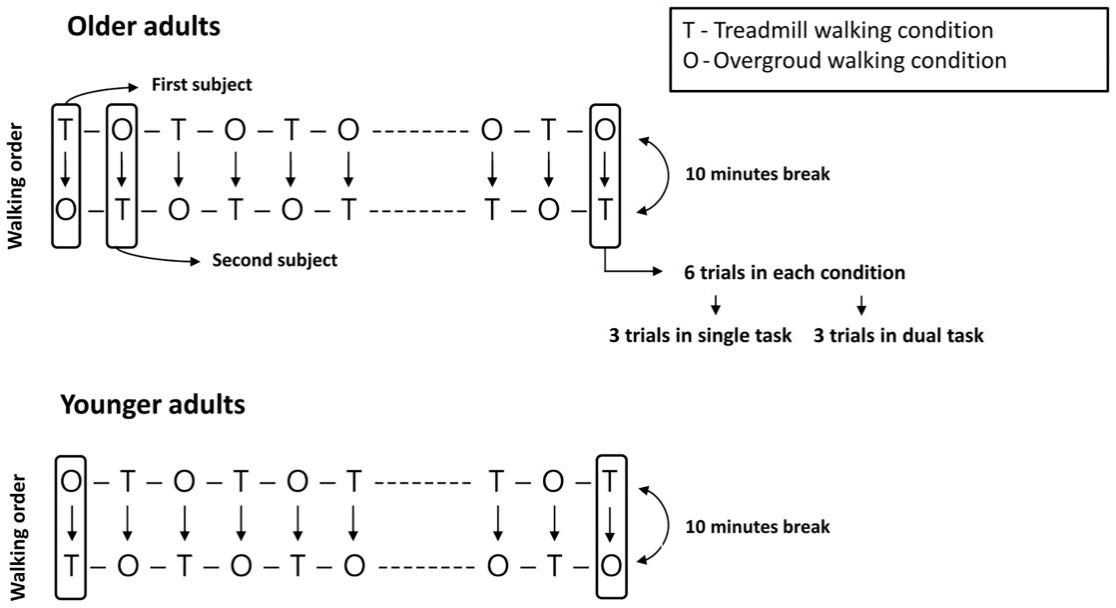
The order of different walking tasks in both older and younger adults. In the older adult groups, the first subject performed treadmill walking as the initial condition, followed by the overground walking condition, whereas in younger adults, the order was reversed. In each group, the second subject’s order was reversed.

For the cognitive task in the single-task condition, the subjects performed serial subtractions of 7 from 3-digit numbers (i.e., 100 and 200) randomly provided by the assessor for 30 seconds, and the correct response (CR), total response (TR), error score (ES) (the number of errors/CR × 100), and response index (RI) (the number of CR/TR × 14) were calculated [9, 21]. This task was first performed with the subjects in a seated position before being re-evaluated in overground and treadmill walking. A dual-task evaluation (i.e., walking with a secondary task) in different conditions was conducted 20 minutes after the cognitive single-task evaluation to avoid any learning effect. The order of the walking conditions for each group was counterbalanced. The first subject in the older group proceeded in overground to treadmill walking order, and in the younger group proceeded in the reverse order, and then the order of conditions of the second subject was reversed depending on the order of the walking condition performed by the first subject in each group (Fig 1). To rule out subject fatigue, which could influence task performance, [33] a 10-min break was provided between the different walking conditions, and the order of dual-task and single-task trials was randomized within each walking condition. There was a break of more than 45 seconds between trials in each condition. The gait assessments lasted for 30 s for each condition. Each subject maintained a quiet position for 30 s before performing the tasks, and tasks started with the instruction "beginning." In the single-task gait, walking proceeded without cognitive tasks, while in the dual-task gait, participants performed a cognitive task (serial subtractions by 7) while walking. The three-digit number between 100 to 200 was presented when walking was initiated. The serial subtraction task during walking was widely used as it induced more PFC activation and was related to executive functions [34, 35]. Furthermore, to minimize task prioritization during the dual-task walking test the subjects were instructed as follows: "distribute your concentration evenly on walking and subtraction tasks" [17]. Subjects performed all walking tasks at their natural walking speed. The subject’s response was simultaneously recorded using a recorder to calculate cognitive task parameters (CR, TR, RI and ES) [36]. During all gait tasks, PFC activation and dual-task performance (gait and cognitive task) were measured during overground and treadmill walking.

### Assessment

#### Dual task performance

OptoGait (Microgate Srl, Bolzano, Italy) was used to evaluate gait performance, which provided excellent reliability and strong validity in overground and treadmill walking tests in healthy and injured adults [37–39]. The gait pattern was analyzed when the subject passed the sensor bars, including light-emitting diodes [40]. This equipment consists of 10 sensor bars (length 1 m). It can be installed in overground and treadmill walking conditions. In the overground walking assessment, sensor bars were installed on both sides with a length of 5 m in the center of a 14-m walkway. In overground walking test, the participant was instructed to walk back and forth for 30 s by crossing the sensor bars in the walkway. At the same time, the straight distance (5m) before the subject made the first turn was processed as a gait analysis period [41]. In treadmill walking test, the sensor bars were positioned on both sides of the treadmill belt matching the height of the treadmill belt in which the subject walks for 30 s [38]. No analysis was done until the subject has reached a preferred speed when starting walking on the treadmill. Afterwards, the evaluation was carried out for 30 seconds at the preferred speed, which was previously measured. The analysis processing period on the treadmill excluded the first and last 5 s to remove acceleration and deceleration phases. The extracted gait parameters in the single-task and dual-task conditions were speed, stride length, gait variability (calculated as [SD stride time/mean stride time] × 100%), cadence, swing phase and double limb support (DLS) time. Here, low stride time variability represented an indicator of automatic and regular walking and a clinical index of gait stability [42]. To assess dual-task gait (gait variability, stride length, speed, cadence, swing phase and DLS) and cognitive performance (TR, CR, and RI), the dual-task cost (DTC) was used as recommended by several studies, [17, 40, 43, 44] with a lower DTC representing good task performance and lower DTI. The ES was not applied to the DTC because there were cases where errors were not found in a single-task situation. The formula is as follows: DTC = (single-task parameter − dual-task parameter)/single-task parameter × 100, with parameter representing either the motor or cognitive task parameter. For gait variability and DLS where a high value indicates poor performance, this formula was reversed.

#### PFC activation

**Fig 2:**
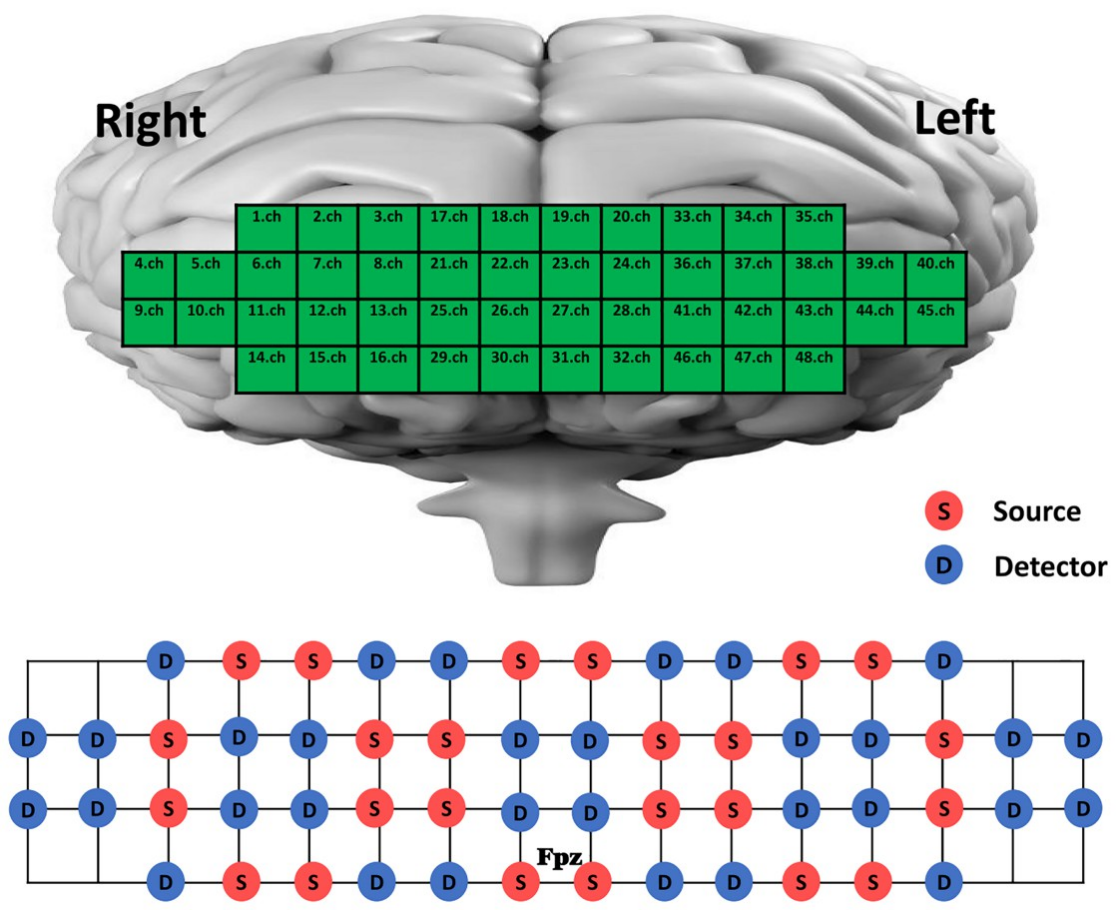
The arrangement of channels in PFC subregions including information on the number and location of source-detector pairs. The numbers represent channels. Ch, channel.

We used a wearable 48-channel fNIRS device (NIRSIT, OBELAB Inc., Seoul, South Korea), which is specialized for PFC activation measurement, [45, 46] compared to the equipment with only 2 to 16 channels used in other studies, [9, 10, 13, 14, 47, 48] as it covers the overall prefrontal area [46, 49]. Therefore, PFC area was divided into the dorsolateral PFC (DLPLF), frontopolar PFC (FPFC), ventrolateral PFC (VLPFC), and the orbital frontal cortex (OFC). The arrangement of the channels is as follows: 1) right DLPFC: 1, 2, 3, 5, 6, 11, 17, and 18; left DLPFC: 19, 20, 33, 34, 35, 38, 39, and 43 channels, 2) right FPFC: 7, 8, 12, 13, 21, 22, 25, and 26; left FPFC: 23, 24, 27, 28, 36, 37, 41, and 42 channels, 3) right VLPFC: 4, 9, and 10; left VLPFC: 40, 44, and 45 channels, and 4) right OFC: 14, 15, 16, 29, and 30; left OFC: 31, 32, 46, 47, and 48 channels (Fig 2) [45, 46]. We used wavelengths of 780 nm and 850 nm with an 8.138-Hz sampling rate, and we set the interoptode distance, including the 24 sources and 32 detectors (Fig 2), to only 3 cm, which could obtain the greater contribution of the cerebral layer than extracerebral layers [46]. In accordance with the localization of the FPz based on the 10–20 international EEG system, this device was placed on the forehead and fixed by an elastic band and calibrated for each subject to eliminate signal saturation provided by biological factors (e.g., skin and hair color, skull thickness). The oxygenated hemoglobin (HbO2) in the prefrontal area was measured during each task under different walking conditions. Deoxygenated hemoglobin (HHb) was not measured based on previous findings that HbO2 directly reflected cortical activity and was more sensitive to locomotion-related activities than HHb [9, 50, 51]. For each trial, the 60-s task duration was divided into a 30-s baseline period, which was measured in quiet standing without any movement as a reference, and a 30 s task period [8, 18, 20, 48, 52]. Hence, we calculated the difference between the baseline and task periods to measure the relative changes in HbO_2_ [9, 48]. Furthermore, to maintain consistency of walking task methods in different walking conditions on the measurement of PFC (e.g., straight walking), the turning section of overground walking test was excluded from the measurement period. To minimize the impact of external factors, such as the surrounding environment, all walking tasks were conducted in a quiet, dimly lit room with only two evaluators and one subject. To exclude the subject’s anticipatory responses, only the middle 20 s per period were used for data processing, excluding the 5 s after the start and 5 s before the end. After one trial (60s) was completed, the subject rested for more than 45 s to ensure the reduced responsiveness of the PFC activation [8]. Additionally, the settled responsiveness was reconfirmed through visual inspection of the time period preceding the subsequent trial. All trials were performed three times, and the results were averaged.

#### Data processing

For data processing, the NIRSIT processing package program based on MATLAB (Version 2019b; MathWorks Inc., Natick, Mass., USA) was used. In the process of converting the raw data extracted from the equipment into optical density, channels with a low signal-to-noise ratio (< 30 dB) were rejected to exclude unreliable or distorted channels due to environmental noise. Furthermore, we processed the signals through the well validated motion artifact reduction algorithm to reduce inappropriate signals caused by head movements during walking and turning [53]. The threshold for the moving standard deviation time series of the algorithm was set at 1 μM [53]. Additionally, a 4^th^-order Butterworth bandpass filter with a low-pass filter at 0.1Hz and high-pass filters at 0.01Hz, respectively, was applied, to eliminate respiration-0.2Hz, heart rate-0.5Hz, Mayer wave-0.1Hz, equipment noise and other unrelated physiological noises [8]. After processing the optical density using the modified Beer-Lambert law, changes in HbO2 were determined for each channel. Baseline correction was applied using the last 5 seconds of the pre-task period as the baseline for each period. To minimize signal contamination by extra-brain artifacts, a multi-channel regression method was done in each PFC subregions [54]. The average HbO_2_ value in each subregion was measured [47–49]. The fNIRS signal processing was conducted based on the signal processing flowchart from a previous study [46]. After the data was processed with the assistance of expert, a visual inspection of the data was also performed once more.

### Statistical analysis

The Shapiro–Wilk test was used to test the normal distribution. The baseline characteristics between groups were compared using the independent t-test, Mann*-*Whitney U test, and chi-square test. Values are presented as mean and standard deviation. To explore the effect of age, walking condition, and task factors on PFC activation, we used a 2 × 2 × 2 mixed-design analysis of variance (ANOVA) with age (older vs. younger adults) as the between-subjects variable and walking condition (overground vs. treadmill) and task (single- vs. dual-task) as the within-subjects variables. We also performed a 2 × 2 mixed ANOVA on dual-task performance with age (older vs. younger adults) and walking condition (overground vs. treadmill) as the between-subjects and within-subjects variables, respectively. A Bonferroni post hoc test was conducted to check for significant interaction effects. Furthermore, the correlation between PFC activation and DTC of dual-task performance was confirmed in various walking conditions using Pearson’s correlation analysis. The significance level was set at p < 0.05. A minimum sample size of 36 subjects was calculated for this study using G * power 3.1, with an effect size of 0.25 and a statistical power of 95%. A sample size of 30 subjects was needed in previous findings with an effect size of 0.96 (Cohen’s d), [13, 22] indicating that the study had a larger sample size compared to other studies examining fNIRS data between younger and older adults [13, 14, 20].

## Results

### Baseline participant characteristics

Table 1 presents the baseline characteristics of the participants. There were no differences in global cognitive function, cognitive performance under single-task conditions, and gait speed under single-task and dual-task conditions between younger and older adults. However, a significant difference was observed in executive function, as assessed by the TMTB (P < 0.001). Additionally, deficits in physical performance, depression, and fear of falling were not found in older adults, as assessed by the SPBB, GDS, and FES-I, with cut*-*off scores of ≤ 9, ≥ 11, and > 23, respectively [29–31]. All older people had no previous falls.

**Table 1:** Baseline characteristics of participants.

Baseline characteristic	Older adults (N = 20)	Younger adults (N = 20)	P
Age, years	64.35 ± 2.74	30.00 ± 3.15	
Sex			
Male/Female	8/12	13/7	0.113
Race			
Asian/Non-Asian	20/0	20/0	-
Education	14.45± 1.67	15.50± 1.96	0.076
Global cognitive function			
MoCA score^a^	27.00 ± 1.75	29.00 ± 2.00	0.053
Executive function			
TMTB, s	110.43 ± 24.81	49.30 ± 12.04	< 0.001
Single-task cognitive performance			
Correct response, n	11.05 ± 3.97	13.10 ± 3.93	0.065
Total response, n	11.38 ±3.91	13.42 ±3.82	0.103
Response index, %	13.57 ± 0.70	13.61 ± 0.61	0.911
Error response, n	0.33 ± 0.56	0.32 ± 0.57	0.812
Single-task gait speed, m/s	1.24 ± 0.15	1.25 ± 0.14	0.813
Dual-task gait speed, m/s	1.17 ± 0.13	1.17 ± 0.17	0.899
Preferred treadmill speed, m/s	1.15 ± 0.13	1.21 ± 0.10	0.125
SPPB score	11.95 ± 0.22	NA	NA
Depression			
GDS score	4.75 ± 2.00	NA	NA
Falling factor			
FES-I score	16.95 ± 5.49	NA	NA
Fall within 6 months			
Yes/No	0/20	NA	NA

FES-I, Falls Efficacy Scale-International; GDS, Geriatric Depression Scale; MoCA, Montreal Cognitive Assessment; SPPB, Short Physical Performance Battery; TMTB, Trail Making Test B,

^a^, applied by Mann-Whitney U test

### Dual-task performance

#### Gait performance

**Fig 3:**
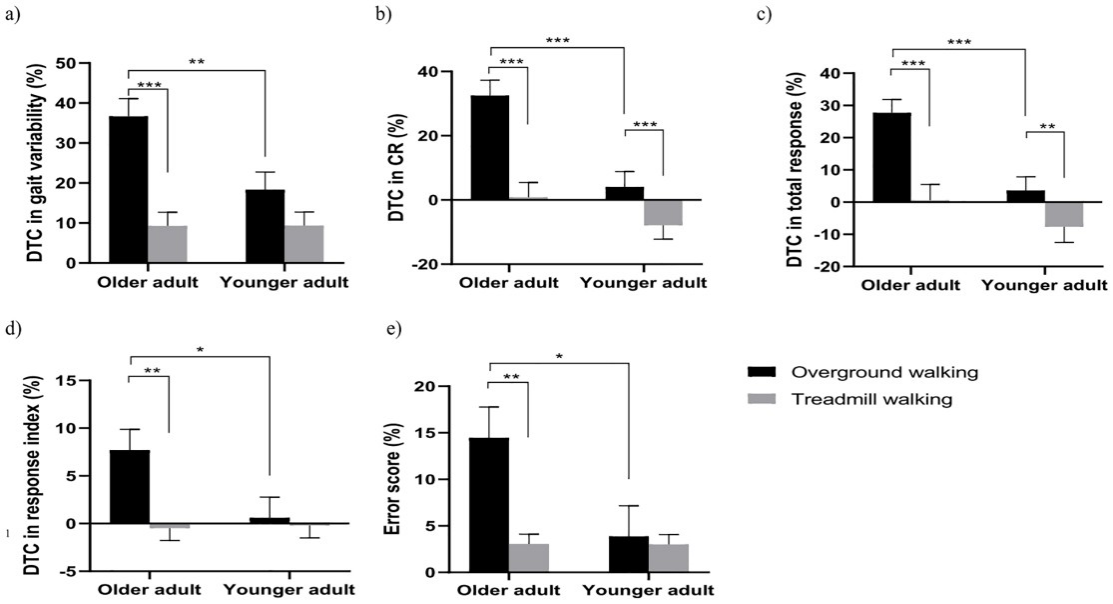
Comparison of DTC in dual-task performance (Mean ± SE). The values of older adults during overground walking were significantly higher than those during treadmill walking, and higher than those during overground walking in younger adults. (a) DTC in gait variability. (b) DTC in CR. (c) DTC in total response. (d) DTC in response index. (e) Error score. CR, correct response; DTC, dual-task cost; *, P < 0.05, **, P < 0.01, ***, P < 0.001.

The main effects for the condition were observed on the DTC gait variability (F (1,38) = 32.365, P < 0.001, partial η2 = 0.461), DTC stride length (F (1,38) = 15.837, P < 0.001, partial η2 = 0.294), DTC speed (F (1,38) = 57.464, P < 0.001, partial η2 = 0.602), DTC swing phase (F (1,38) = 38.936, P < 0.001, partial η2 = 0.506) and DTC DLS (F (1,38) = 69.121, P < 0.001, partial η2 = 0.645). The post-hoc test revealed the values were higher in overground walking than those in treadmill walking. An interaction effect between condition and age was observed in DTC gait variability (F (1,38) = 8.317, P = 0.006, partial η2 = 0.180). The post-hoc test revealed that the DTC gait variability of older adults during overground walking was higher than that during treadmill walking, and higher than that during overground walking in younger adults (Fig 3).

#### Cognitive task performance

The main effects for age were found on DTC of CR (F(1,38) = 8.615, P = 0.006, partial η2 = 0.185), TR (F(1,38) = 7.324, P = 0.010, partial η2 = 0.162), and ES (F(1,38) = 4.642, P = 0.038, partial η2 = 0.109), and for condition were observed in DTC of CR (F(1,38) = 112.121, P < 0.001, partial η2 = 0.747), TR (F(1,38) = 60.829, P < 0.001, partial η2 = 0.615), RI (F(1,38) = 7.337, P = 0.010, partial η2 = 0.162), and ES (F(1,38) = 6.487, P = 0.015, partial η2 = 0.146). The interaction effects between the two factors were found in all cognitive variables such as the DTC of CR (F(1,38) = 23.035, P < 0.001, partial η2 = 0.377), TR (F(1,38) = 10.421, P = 0.003, partial η2 = 0.215), RI (F(1,38) = 4.960, P = 0.032, partial η2 = 0.115), and ES (F(1,38) = 4.795, P = 0.035, partial η2 = 0.112) (Table 2). The post-hoc test results indicated that, compared to younger adults, older adults exhibited higher DTC of CR, TR, and RI, as well as higher ES during overground walking. Additionally, older adults showed higher values for these parameters during overground walking compared to treadmill walking (Fig 3).

**Table 2:** Comparison of dual task performance.

	Older adults (N = 20)		Younger adults (N = 20)		Main effect		Interaction effect
	Over-ground walking	Treadmill walking	Over-ground walking	Treadmill walking	Condition	Age	Condition by Age
DTC in motor task							
Gait variability, %	36.70 ± 22.49	9.28 ± 15.88^a^	18.35 ± 16.36^d^	9.34 ± 14.54	< 0.001	0.052	0.006
Stride, %	5.04 ± 3.55	2.49 ± 6.81^b^	1.00 ± 3.92	-1.56 ± 5.95^b^	< 0.001	0.058	0.992
Speed, %	7.47 ± 5.47	0.67 ± 1.62^a^	4.99 ± 2.81	0.77 ± 1.57^a^	< 0.001	0.116	0.085
Cadence, %	3.01 ± 4.87	1.64 ± 8.62	4.65 ± 3.30	2.09 ± 3.37	0.109	0.408	0.623
Swing phase, %	4.84 ± 3.16	1.25 ± 2.20^a^	4.63 ± 4.19	-0.02 ± 2.28^a^	< 0.001	0.301	0.428
Double limb support, %	7.33 ± 2.61	1.49 ± 2.24^a^	4.94 ± 2.74^d^	1.31 ± 1.85^a^	< 0.001	0.013	0.060
DTC in cognitive task							
Correct response, %	32.51 ± 24.11	0.80 ± 23.63^a^	4.07 ± 18.12^c^	-7.86 ± 17.32^a^	< 0.001	0.006	< 0.001
Total response, %	27.72 ± 21.16	0.56 ± 26.78 ^a^	3.65 ± 15.18^c^	-7.61 ± 15.72 ^b^	< 0.001	0.010	0.003
Response index, %	7.71 ± 12.82	-0.47 ± 6.07 ^b^	0.60 ± 4.87^e^	-0.20 ± 5.62	0.010	0.082	0.032
Error score, %	14.48 ± 19.65	3.05 ± 4.28 ^b^	3.87 ± 6.79^e^	3.00 ± 5.21	0.015	0.038	0.035

DTC, dual task cost,

^a^ p < 0.001, indicating a significant difference between overground and treadmill walking,

^b^ p < 0.01, indicating a significant difference between overground and treadmill walking,

^c^ p < 0.001, indicating a significant difference between groups in overground walking,

^d^ p < 0.01, indicating a significant difference between groups in overground walking,

^e^ p < 0.05, indicating a significant difference between groups in overground walking.

### PFC activation

**Fig 4:**
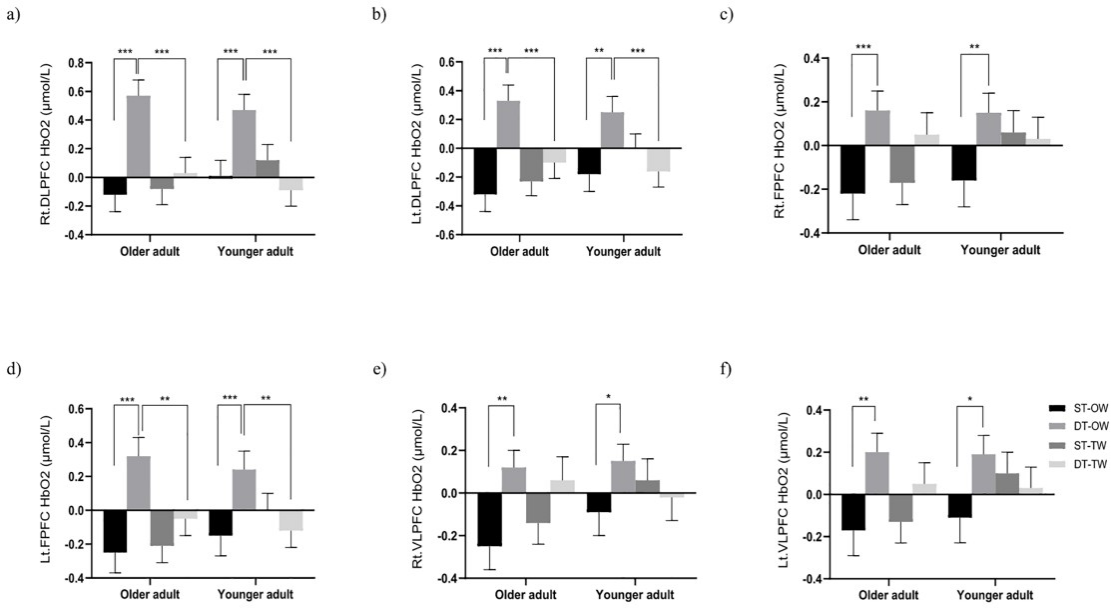
Comparison of PFC activation (Mean ± SE). The values of PFC activation during dual-task overground walking were significantly higher than those during single-task overground walking, and higher than those during dual-task treadmill walking. (a) Right DLPFC activation. (b) Left DLPF activation. (c) Right FPFC activation. (d) Left FPFC activation. (e) Right VLPFC activation. (f) Left VLPFC activation. PFC, prefrontal cortex; DLPFC, dorsolateral PFC; FPFC, frontopolar PFC; VLPFC, ventrolateral PFC; HbO_2_, oxygenated hemoglobin; ST, single-task; DT, dual-task; OW, overground walking; TW, treadmill walking; *, P < 0.05, **, P < 0.01, ***, P < 0.001.

The main effect of condition was observed on right DLPFC (F (1,38) = 19.006, P < 0.001, partial η2 = 0.333) and left DLPFC (F (1,38) = 7.337, P = 0.010, partial η2 = 0.162). The main effect of task was observed on right DLPFC (F (1,38) = 15.258, P < 0.001, partial η2 = 0.286), left DLPFC (F (1,38) = 18.402, P < 0.001, partial η2 = 0.326), right FPFC (F (1,38) = 15.420, P < 0.001, partial η2 = 0.289), left FPFC (F (1,38) = 15.848, P < 0.001, partial η2 = 0.294), right VLPFC (F (1,38) = 10.190, P = 0.003, partial η2 = 0.211), and left VLPFC (F (1,38) = 12.200, P = 0.001, partial η2 = 0.243). This indicated that PFC activation during treadmill walking was lower than that during overground walking, and dual-tasking led to higher PFC activation compared to single-tasking. In addition, the interaction effect of condition by task was found in right DLPFC (F (1,38) = 35.655, P < 0.001, partial η2 = 0.484), left DLPFC (F (1,38) = 19.799, P < 0.001, partial η2 = 0.343), and left FPFC (F (1,38) = 20.761, P < 0.001, partial η2 = 0.353). The post-hoc tests showed that in both older and younger adults, PFC activation during dual-task overground walking was higher than during single-task overground walking. However, no significant difference in activation between tasks was observed during the treadmill condition. Furthermore, dual-task activation during treadmill walking was significantly lower than that during overground walking (Fig 4). No effect of interaction by age by condition by task was observed. Furthermore, a positive correlation was observed between PFC activation (both DLPFCs and both FPFCs) and DTC during overground walking, but not during treadmill walking (Table 3).

**Table 3:** Relationship between PFC activation and performance during dual-task overground walking.

	r	P-value	95% CI	r	P-value	95% CI
Overground walking						
	**Right side DLPFC activation**			**Left side DLPFC activation**		
Gait variability, %	0.421	0.007	-0.001, 0.725	0.453	0.003	0.012, 0.755
Correct response, %	0.363	0.021	0.016, 0.622	0.353	0.025	0.023, 0.590
Total answer %	0.383	0.015	0.006, 0.639	0.366	0.020	0.035, 0.606
Response index, %	0.411	0.008	-0.038, 0.647	0.422	0.007	-0.003, 0.670
Error score, %	0.360	0.022	-0.055, 0.580	0.373	0.018	-0.023, 0.582
	**Right side FPFC activation**			**Left side FPFC activation**		
Gait variability, %	-.0137	0.400	-0.484, 0.201	0.012	0.984	-0.380, 0.367
Correct response, %	0.329	0.038	0.086, 0.549	0.293	0.067	-0.091, 0.622
Total answer %	0.341	0.031	0.094, 0.575	0.366	0.020	-0.014, 0.660
Response index, %	0.225	0.163	-0.054, 0.542	0.295	0.064	-0.153, 0.558
Error score, %	0.079	0.628	-0.119, 0.383	0.369	0.019	0.024, 0.589

PFC, prefrontal cortex, DLPFC, dorsolateral PFC, FPFC, frontopolar PFC. Both DLPFCs and FPFCs are significantly correlated with dual task performance during overground walking, but not during treadmill walking.

## Discussion

To our knowledge, this is the first study to determine and compare PFC activation and DTC during dual-tasking under different conditions among younger and older adults. Our findings suggest that dual-task treadmill walking leads to lower DTC and lower PFC activation compared to dual-task overground walking. Furthermore, differences in DTC in motor and cognitive tasks between older and younger adults, as well as a higher dual-task PFC activation compared to single-task activation were found only in overground walking.

### Dual-task performance

During overground walking, older adults exhibited lower dual-task performance in gait and cognitive task performance, compared to younger adults. This suggests that age-related decline in gait automaticity may affect dual-task walking ability, consistent with previous findings [9, 14]. However, there was no difference in dual-task performance between younger and older adults during treadmill walking and dual-task treadmill walking improved the performance of older adults to the level of younger adults. In a study by Penati et al. [16], dual-task treadmill walking led to a higher cognitive task performance than overground walking. That is, the poorer dual-task performance during overground walking may be due to its greater susceptibility to the effects of DTI. Another previous study has shown that the higher DTC on motor and cognitive performance observed during overground walking was reduced when walking on a treadmill [17]. This is due to the environmental characteristics of the treadmill where externally generated cues (e.g., constant backward treadmill belt) artificially prevent the altered gait pattern caused by DTI, such as decreased speed and stride length, and increase gait automaticity through rhythmic movement [16, 40]. Both gait variability and cognitive performance, indicating automaticity, [40, 55] may be compensated by the influence of the treadmill for older adults; thus, no significant differences between groups would be observed on treadmill walking. This mechanism effect in treadmill walking reduces the attention required for walking during dual-tasking, thereby allocating more attention to the secondary task (cognitive task), leading to superior cognitive processing speed, accuracy, and lower error [16, 17].

### PFC activation

Both older and younger adults had similar patterns in PFC activation in overground and treadmill walking, and they showed high PFC activation during dual-task in overground walking compared to single-task overground walking, revealing similar findings to those of previous studies [12, 52]. However, no difference in PFC activation was found in the treadmill condition, and dual-task treadmill walking showed a lower level of PFC compared to dual-task overground walking. Walking involves two main pathways: the direct locomotor pathway and the executive motor pathway. The direct locomotor pathway transmits signals from the primary motor cortex directly to the spinal network, bypassing the basal ganglia and brainstem locomotor regions. This pathway is primarily activated during normal gait, requiring relatively fewer attentional resources (PFC resource) [8]. On the other hand, the executive motor pathway involves the transmission of signals through the PFC to the basal ganglia, subthalamic, and mesencephalic locomotor regions. This pathway is typically used to compensate for deficits in gait automaticity with more attentional resources in difficult conditions, including dual-task situations and various pathological and aging conditions [1, 8]. That is, the decrease in PFC activation during dual-task walking on a treadmill may indicate the utilization of fewer attentional resources and an improvement in automaticity [8, 19, 23, 56]. Stuart et al. [22] found that older adults showed activation in the premotor, supplementary motor, and primary motor areas during dual-task treadmill walking, but without PFC activation [13]. In previous study, it was found that improved automaticity during dual-task walking on treadmills reduced the activation of the PFC in older adults [57]. Dual-task treadmill walking has been shown to maintain task performance while reducing PFC activation compared to overground walking [56]. In addition, the results of the correlation analysis showed that lower automaticity parameters in overground walking only were associated with higher PFC activation, but not during treadmill walking. However, no differences were observed in PFC levels between the two groups in the study, possibly because the older adults in the study were younger than those in other similar studies (mean age, <65 years vs. ≥70 years) [12, 58]. This suggests that there may have been less age-related decline in this sample. As shown, the present study assessed aging progression using the TMTB. However, despite this measure, the older adults in the study did not differ significantly from the younger adults in terms of global cognitive function, physical function, and gait ability, as measured by the MoCA, SPPB, and walking speed, respectively. Additionally, the older adults had no indications of depression or fear of falling. These findings suggest that the decrease in physical function and gait ability with aging may not be severe enough to require compensatory PFC overuse in this group of older adults compared to younger adults. Furthermore, a study comparing older adults with advanced aging (77–82 years) to those with less advanced aging (61–70 years) found evidence of age-related over-recruitment in PFC in the former group [59]. This study had several limitations. Firstly, the relatively small sample size made it difficult to generalize the results, despite the study’s justification. Although sample size calculation was carried out, obtaining an appropriate effect size was limited due to the lack of comparable studies. Secondly, the study only included right-handed participants, which may limit the generalizability of the results to left-handed individuals. Additionally, since the study design only targeted PFC measurements, including measurements from other brain regions would provide a clearer interpretation of the results. Moreover, the use of brain imaging techniques with high spatial resolution could have been helpful in determining the activation patterns of the brain. The older participants were not further assigned to different age groups, which is necessary to reveal the corresponding aging stages, including less advanced and advanced aging. Finally, the analysis period was different between the two walking conditions in the gait performance evaluation, but a gait analyzer was used that could be applied to both treadmill and overground walking conditions to match the study design as closely as possible [37–39, 60]. To investigate the effects of different walking conditions on dual-task performance and PFC activation in both younger and older adults, it is crucial to examine variables with varying speed factors (slow versus fast) and different cognitive tasks in future studies. Moreover, to support the results of this study, randomized controlled experimental studies are essential, not just cross-sectional ones.

This first study will help demonstrate the effects of various walking environments on dual task performance and task-related PFC activation in younger and older adults, and these findings will help design studies involving dual-task walking, especially for older adults with reduced gait automaticity.

### Conclusion

When performing dual-task walking on a treadmill compared to overground walking, different levels of dual-task performance and PFC activity are observed. Older adults are able to maintain similar levels of dual-task performance as younger adults while walking on a treadmill, with PFC activation reduced due to automaticity induced by the treadmill. This suggests that older adults who exhibit low dual-task performance during overground walking may be able to improve their performance while walking on a treadmill with fewer attentional resources required.

## Abbreviations

CR: correct response
DLPFC: dorsolateral PFC
DLS: double limb support
DTC: dual-task cost
DTI: dual-task interference
ES: error score
FES-I: Falls Efficacy Scale-International
fNIRS: functional near-infrared spectroscopy
FPFC: frontopolar PFC
GDS: Geriatric Depression Scale
HbO_2_: oxygenated hemoglobin
HHb: deoxygenated hemoglobin
MoCA: Montreal Cognitive Assessment
OFC: orbital frontal cortex
PFC: prefrontal cortex
RI: response index
SPBB: Short Physical Performance Battery
TMTB: Trail Making Test B
TR: total response
VLPFC: ventrolateral PFC
